# Carotid body: an emerging target for cardiometabolic co‐morbidities

**DOI:** 10.1113/EP090090

**Published:** 2023-03-30

**Authors:** Pratik Thakkar, Audrys G. Pauza, David Murphy, Julian F. R. Paton

**Affiliations:** ^1^ Manaaki Manawa – the Centre for Heart Research, Department of Physiology, Faculty of Medical and Health Sciences University of Auckland Auckland New Zealand; ^2^ Molecular Neuroendocrinology Research Group, Bristol Medical School: Translational Health Sciences University of Bristol Bristol UK

**Keywords:** carotid body, diabetes, glucagon‐like peptide‐1, high blood pressure, sympathetic nerve activity

## Abstract

The maintenance of glucose homeostasis is obligatory for health and survival. It relies on peripheral glucose sensing and signalling between the brain and peripheral organs via hormonal and neural responses that restore euglycaemia. Failure of these mechanisms causes hyperglycaemia or diabetes. Current anti‐diabetic medications control blood glucose but many patients remain with hyperglycemic condition. Diabetes is often associated with hypertension; the latter is more difficult to control in hyperglycaemic conditions. We ask whether a better understanding of the regulatory mechanisms of glucose control could improve treatment of both diabetes and hypertension when they co‐exist. With the involvement of the carotid body (CB) in glucose sensing, metabolic regulation and control of sympathetic nerve activity, we consider the CB as a potential treatment target for both diabetes and hypertension. We provide an update on the role of the CB in glucose sensing and glucose homeostasis. Physiologically, hypoglycaemia stimulates the release of hormones such as glucagon and adrenaline, which mobilize or synthesize glucose; however, these counter‐regulatory responses were markedly attenuated after denervation of the CBs in animals. Also, CB denervation prevents and reverses insulin resistance and glucose intolerance. We discuss the CB as a metabolic regulator (not just a sensor of blood gases) and consider recent evidence of novel ‘metabolic’ receptors within the CB and putative signalling peptides that may control glucose homeostasis via modulation of the sympathetic nervous system. The evidence presented may inform future clinical strategies in the treatment of patients with both diabetes and hypertension, which may include the CB.

## Abstract (highlights)

- **What is the topic of this review?**
  Regarding the global metabolic syndrome crisis, this review focuses on common mechanisms for high blood sugar and high blood pressure. Connections are made between the homeostatic regulation of blood pressure and blood sugar and their dysregulation to reveal signalling mechanisms converging on the carotid body.
- **What advances does it highlight?**
  The carotid body plays a major part in the generation of excessive sympathetic activity in diabetes and also underpins diabetic hypertension. As treatment of diabetic hypertension is notoriously difficult, we propose that novel receptors within the carotid body may provide a novel treatment strategy.

## 1 INTRODUCTION

The maintenance of blood glucose homeostasis is essential for good health and wellbeing. If glucose rises (hyperglycaemia) it is toxic to peripheral nerves, eyes and blood vessels as well as increasing the risk of heart attacks, stroke and kidney failure (Bourne et al., 2013; Sarwar et al., 2010). The escalating global tsunami of obesity and metabolic disorders, which include diabetes, represents a major global health threat of the 21st century. In Aotearoa/New Zealand over 263,000 people or 5.4% of the population have diabetes, forming a global epicentre (Ministry of Health New Zealand, 2020). According to the World Health Organization, it is estimated that globally 422 million people suffer from diabetes, and of these approximately 1.6 million people die from the illness per year (World Health Organization, 2022). Most alarmingly is that this figure is predicted to rise by almost 50% over the next 25 years (Saeedi et al., 2019).

A better understanding of the regulatory mechanisms of metabolic control could significantly improve the treatment of diabetes and inter‐related cardiovascular comorbidities. In mammals, glucose homeostasis is controlled by a highly coordinated glucose‐sensing mechanism with multiple effector systems. Detection of a change in the level of plasma glucose is ensured by sensors located at several anatomically distinct sites, which work in concert to maintain glucose homeostasis. These sensors are located in the brain, intestine, portal vein and pancreas (Ruud et al., 2017). Emerging evidence suggests that the carotid body (CB), which is well known for responding to changes in blood gases, can also sense plasma glucose and regulate metabolic mechanisms (Gao et al., 2014; Ribeiro et al., 2013; Thompson et al., 2016), making this organ a truly multi‐modal sensor. Herein, we explore the different sensing mechanisms controlling glucose homeostasis and how these may incorporate the CB. We define how reflexes emanating from the CB would operate to reduce blood glucose but may also simultaneously improve associated cardiovascular comorbidities, such as high blood pressure. We discuss the presence and putative function of two G‐protein‐coupled receptors that play significant roles in well‐established mechanisms of glucose homeostasis and have been identified within the CB. Such information could offer novel potential therapeutic targets within the CB to further improve the future treatment of the metabolic syndrome.

## 2 NEURONAL MECHANISMS OF GLUCOSE SENSING

**FIGURE 1:**
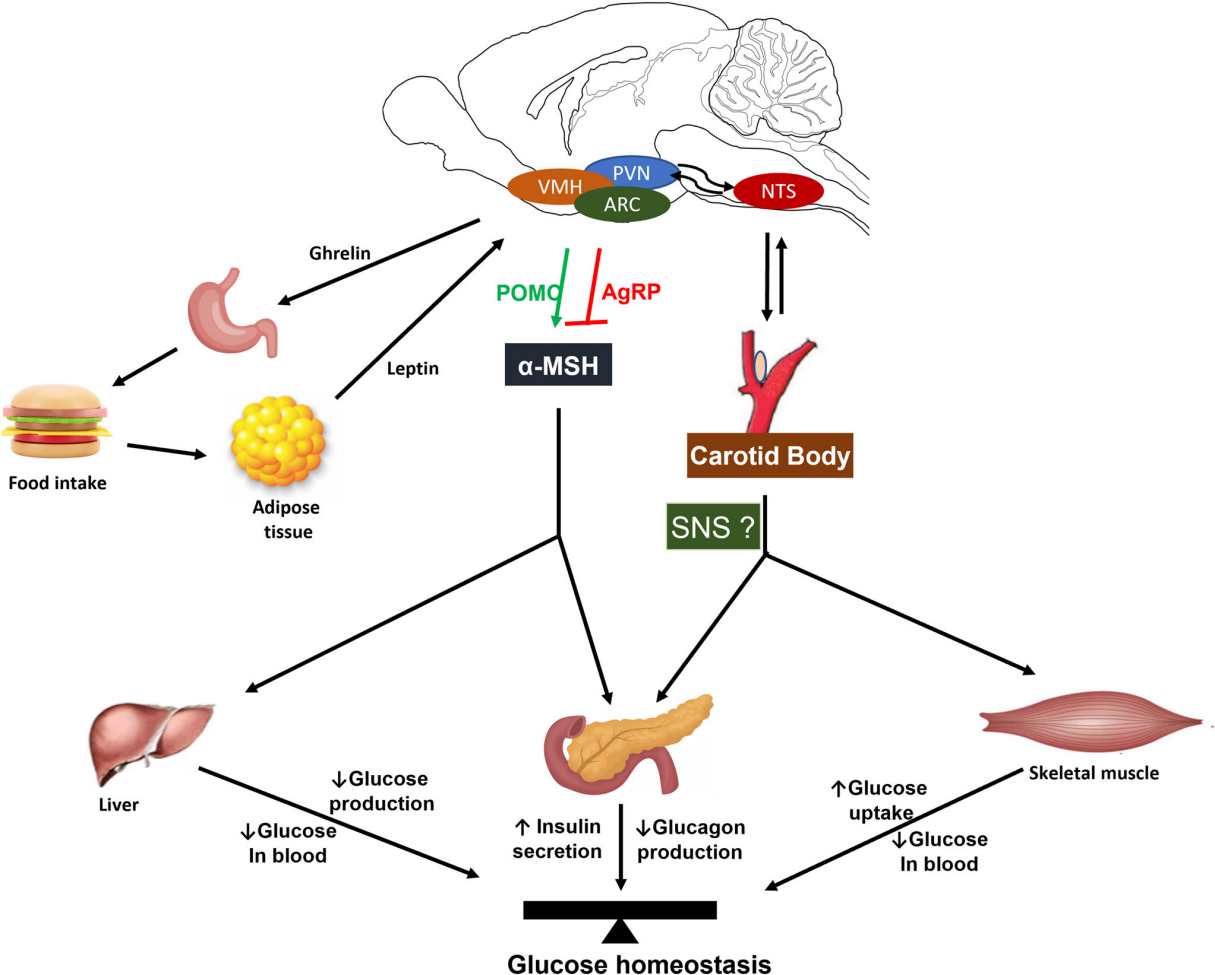
Proposed central and peripheral mechanisms of glucose homeostasis. The brain senses peripheral metabolic signals through both nutrients (glucose) and hormones (ghrelin, insulin, leptin) and regulates glucose metabolism via neuronal signalling from the hypothalamus and brainstem. Specialized neuronal networks such as agouti‐related protein (AGRP) and proopiomelanocortin (POMC)‐producing neurons in the hypothalamic arcuate nucleus and ventromedial region coordinate adaptive changes in response to altered metabolic conditions via changes in autonomic and hormonal function. The brainstem participates in homeostatic regulation of glucose homeostasis and this includes inputs from the carotid body (CB). Leptin increases blood pressure acting both centrally in the hypothalamus and peripherally by enhanced activity of the carotid body. Hence, the CB is an important modulator of information for the gut–brain–autonomic axis that modulates pancreatic insulin/glucagon secretion and skeletal muscle glucose uptake. ARC, arcuate nucleus; α‐MSH, α‐melanocyte stimulating hormone; NTS, nucleus of the solitary tract; PVN, paraventricular nucleus; SNS, sympathetic nervous system; VMH, ventromedial hypothalamus. Schematic representation based on data including those from Shimazu and Minokoshi (2017) and Shin et al. (2019).

To better understand how and when the CBs might sense glucose, it is useful to briefly discuss the mechanisms of glucose sensing in the brain and the pancreas. Glucose‐sensing neurons are localized mainly in the hypothalamic region and brainstem and play a critical role in glucose homeostasis due to their ability to sense, integrate and respond to changes in circulating glucose (Figure 1) (Myers & Olson, 2012). Responses are coordinated via both the autonomic nervous (ANS) and endocrine systems, which control the initiation and termination of feeding (satiety) as well as energy homeostasis (plasma glucose). The hypothalamus contains two types of specialized glucose‐sensing neurons: glucose‐excited (HGE) and glucose‐inhibited (HGI) neurons (Song et al., 2001). HGE neurons increase their electrical activity when extracellular glucose levels rise, while HGI neurons increase their electrical activity when the extracellular glucose levels decrease (Routh et al., 2014). These neurons are within the mediobasal hypothalamic nuclei and include the arcuate (ARC) and ventromedial hypothalamic nuclei, paraventricular nucleus (PVN) (Gao et al., 2008) and brainstem (e.g., the nucleus tractus solitarii) (Holt et al., 2019). The glucose level in the brain usually is 2 mM, which is two to three times less than blood levels (fasting: 4 mM; post‐prandial 7 mM) (Langlet et al., 2013). Changes in blood glucose levels recruit these neurons to activate a range of homeostatic mechanisms (Song et al., 2001)

Glucose‐sensing neurons of the ventromedial hypothalamus (VMH) also have an important role in regulating glucose homeostasis by altering secretion of pancreatic hormones, such as insulin and glucagon (Stanley et al., 2016). These VMH neurons maintain blood glucose levels within a tight range (0.7–2.5 mM) due to their relatively higher sensitivity to glucose than the ARC neurons (Ruud et al., 2017), which is mediated by the sympathetic nervous system (SNS) innervation of the liver, brown fat and skeletal muscle as well as neuroendocrine mechanisms (Figure 1) (Shimazu & Minokoshi, 2017; Sudo et al., 1991). In addition, feedback from adipose‐released leptin acts on the VMH to increase glucose uptake via responses mediated by the SNS (Shimazu & Minokoshi, 2017). This results in lower hepatic glucose production and increased peripheral glucose uptake (Chan et al., 2008). Sympathetic activation is a major mechanism following VMH neuron stimulation for increasing the uptake of glucose in peripheral tissues (Shimazu et al., 1991, 1998); such a response is prevented after sympathetic denervation or chemical sympathectomy (Minokoshi et al., 1994; Sudo et al., 1991). All told, these findings reveal a direct involvement of the SNS in glucose homeostasis, and this is likely to be relatively sensitive as it is activated during modest perturbations in plasma glucose.

The ARC nucleus plays a fundamental role in energy homeostasis. It contains two main neuronal populations: (i) neuropeptide Y (NPY)/agouti‐related protein (AgRP)‐ and pro‐opiomelanocortin (POMC)‐containing neurons that regulate both energy and glucose homeostasis in an opposing manner (Ollmann et al., 1997; Shutter et al., 1997). When circulating glucose levels are elevated, HGE POMC neurons are activated during positive energy balance, leading to the secretion of α‐melanocyte‐stimulating hormone (α‐MSH). α‐MSH binds to melanocortin receptors in the PVN and induces both an anorexigenic effect and regulation of glucose metabolism via the pancreas (Mountjoy et al., 1994; Schwartz et al., 2000). In contrast, during food deprivation and reduced circulating glucose levels, NPY/AgRP neurons are activated and inhibit the melanocortin pathway (Nijenhuis et al., 2001). The ability of these two populations of ARC neurons to alter body metabolism is due to their sensitivity to several circulating hormones (e.g., ghrelin, leptin, insulin) as well as blood glucose (Roh et al., 2016; Shimazu & Minokoshi, 2017). Interestingly, ARC glucose‐excited POMC neurons show a similar glucose‐sensing mechanism to that observed in pancreatic β‐cells.

## 3 PANCREATIC MECHANISMS OF GLUCOSE‐SENSING

Glucose‐dependent release of insulin by pancreatic β‐cells is a key physiological event contributing to glucose homeostasis. Insulin release is stimulated after ingesting a carbohydrate‐containing meal leading to raised blood glucose. The latter acts on the pancreas to release insulin into the blood and accelerate cellular glucose uptake and enhance metabolic glucose disposal and storage (Schuit, 1996). Generally, insulin output between meals is balanced by counteracting hormones such as glucagon and adrenaline to prevent overcompensation and hypoglycaemia. The glucose‐sensing mechanism of pancreatic β‐cells can be divided into two components: (i) glucose entry and metabolism and (ii) insulin secretion.

Glucose can quickly circulate across the β‐cell membrane with help from glucose transporter‐2 (GLUT2) (Newgard & McGarry, 1995); it is then phosphorylated to glucose‐6‐phospate by the high‐*K*_M_ glucokinase (hexokinase IV) (De Vos et al., 1995), which is considered to be the ‘glucose sensor’ in the pancreatic β‐cells. Once glucose is phosphorylated, it is metabolized by glycolysis to produce pyruvate, NADH and ATP, where pyruvate is a vital substrate for the tricarboxylic acid (TCA) cycle in mitochondria (Zawalich & Zawalich, 1997). Both cytosolic pyruvate and NADH enter the mitochondria (Eto et al., 1999). In addition to cytosolic NADH, NADH is also produced from pyruvate in the mitochondria by the TCA cycle. Both cytosolic and mitochondrial sources of NADH stimulate the electron transport chain to pump H^+^ ions out of the mitochondrial matrix, hyperpolarizing the inner mitochondrial membrane to increase mitochondrial Ca^2+^ transportation (Eto et al., 1999). This process augments Ca^2+^‐dependent dehydrogenase activity and further increases the production of NADH and ATP from the TCA cycle (Hansford, 1991). This increase in intracellular ATP and especially the ATP‐to‐ADP ratio in pancreatic β‐cells leads to closure of K_ATP_ channels and membrane depolarization (MacDonald et al., 2005). This activates voltage‐dependent Ca^2+^ channels (VDCCs), allowing influx of Ca^2+^, which triggers exocytosis of insulin into the blood stream.

## 4 THE CB AND GLUCOSE SENSING

Homeostasis is never without multiple parallel mechanisms and the sensing of blood glucose is no exception. These parallel mechanisms have different sensitivities as described above but are likely to operate over different time frames such as the time to respond to a glucose perturbation and the duration of the response(s) evoked. The mechanisms of glucose sensing by the ARC, POMC, VMH and pancreas and the evoked neuroendocrine mechanisms described above operate over a time frame of minutes. Indeed, insulin release is 1.4 nmol/min with regular bursts occurring every 4 min releasing 0.4 nmol/min in humans (Fu et al., 2013; Kashyap et al., 2003). This invites the question of whether there are mechanisms that operate more immediately and facilitate these other slower to respond mechanisms. We propose this role to be accommodated by the CB.

The CB is a small organ located bilaterally at the bifurcation of the common carotid artery. Although the CB has been defined as a sensor of blood gases (hypoxia and hypercapnia) and blood pH, it is now considered as a metabolic sensor (Cracchiolo et al., 2019). For example, the CB was shown to respond to high blood glucose (Gao et al., 2014), elevated insulin (Ribeiro et al., 2013) and raised adrenaline (Thompson et al., 2016); however, the molecular mechanisms for sensing glucose are not fully established, as described below. Thus, current evidence supports the notion that CB has a fundamental role in glucose homeostasis. Any change in CB function, which includes either hyper‐reflexia and/or hypertonicity as found in cardiovascular diseases (Pijacka et al., 2016; Schultz & Marcus, 2012), may therefore contribute to the pathogenesis of diabetes. Given this, the opportunity presents itself that the CB may be a new target to ameliorate diabetes.

## 5 MOLECULAR MECHANISM FOR GLUCOSE SENSING BY THE CB

Multiple studies have reported glucose sensing mechanisms by the CB from animals and humans (Fitzgerald et al., 2009; García‐Fernández et al., 2007; Pardal & López‐Barneo, 2002; Ward et al., 2009; Zhang et al., 2007). In animals, CB cells have insulin receptors and respond to increases in insulin levels (Bin‐Jaliah et al., 2004; Ribeiro et al., 2013). Koyama et al. showed that CB resection in dogs impaired (i) the counter‐regulatory response to insulin‐induced hypoglycaemia and (ii) the exercise‐mediated induction of glucagon and noradrenaline, compromising the maintenance of blood glucose (Koyama et al., 2001, 2000). Past studies report that the CB responds to both low and high glucose levels in the blood and drives counter‐regulatory mechanisms in glucose homeostasis (Gao et al., 2014).

**FIGURE 2:**
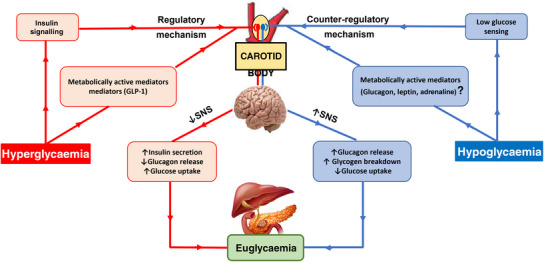
Metabolic sensory signalling to the carotid body (CB). The CB plays a significant role in mediating compensatory responses for glucose homeostasis. During a hypoglycaemic state, glomus cells sense low blood glucose levels and trigger increases in sympathetic nerve activity along with specific metabolic hormones, ultimately increasing glucose production via glucagon‐mediated glycogen breakdown. Hyperglycaemia induces increases in circulating insulin and GLP1 that act on the CB that, via the SNS, stimulates a number of regulatory hormones to compensate for raised glucose levels.

In the presence of hypoglycaemia, in vitro studies of glomus cells demonstrated membrane depolarization (carried preferentially by Na^+^ ions) that inhibited voltage‐gated outward K^+^ current causing increases in Ca^2+^ influx observed in rats (García‐Fernández et al., 2007; Zhang et al., 2007), cats (Fitzgerald et al., 2009) and human (Ortega‐Sáenz et al., 2013). GLUT2 and glucokinase, which are important for detecting high glucose, were not important for low glucose detection in the CB (García‐Fernández et al., 2007; Thorens, 2015; Thorens et al., 1988). Additionally, redox regulation of Ca^2+^ channels is an important regulator of glucose metabolism (Wyatt et al., 1994). In hypoglycaemic condition, intracellular increases in Ca^2+^ leads to exocytosis of neurotransmitter release that triggers a reflex increase in sympathetic nerve activity causing a counter‐regulatory *increase* in blood glucose levels (Figure 2) (Fitzgerald et al., 2009; Zhang et al., 2007). Carpenter and Peers reported that when cells are exposed to hypoglycaemic solution, the Na^+^ current played a major role in the chemo‐transduction process of glomus cells. Na^+^ ions set the membrane potential near the threshold for opening of Ca^2+^ channels, which contributes to the counter‐regulatory mechanisms for glucose homeostasis (Carpenter & Peers, 2001). However, the sensitivity of the CB to low glucose has not been confirmed by other in vivo and in vitro animal and human studies, and it has been proposed that other metabolic factors secondary to hypoglycaemia may provide the stimulus for the counter‐regulatory mechanisms of the CB in glucose homeostasis (Bin‐Jaliah et al., 2004; Gallego‐Martin et al., 2012; Ward et al., 2009).

We now turn our attention to the question of the mechanism(s) by which the CBs respond to high glucose. Previous studies indicate that the GLUT2 transporter and glucokinase are the major molecules expressed in gut, pancreas and hypothalamus that provide the basis for hyperglycaemic sensitivity (Schuit et al., 2001; Thorens et al., 1988); whether they contribute or other Glut genes involve in the CB remains unknown. The study involved acute hyperglycaemia (25 mM) in an isolated CB–carotid sinus nerve preparation *ex vivo*, which did not alter carotid sinus nerve activity (Conde et al., 2018). We raise the question as to whether the glucose needs to be infused into the CB via the arterial system, rather than being superfused to see an increase in afferent output. Nevertheless, Ribeiro and colleagues reported that insulin resistance and hypertension produced by hypercaloric diets are completely prevented by bilateral carotid sinus nerve resection. This result supports the concept of the CB's involvement in insulin regulation (Ribeiro et al., 2013). Moreover, hyperglycaemia increased the hypoxic ventilatory response probably triggered by the action of increased circulating insulin on the CB (Ward et al., 2007). The latter is an example of how glucose sensitivity may be interpreted, but in reality the apparent effect of glucose is indirect and mediated by release of secondary metabolic molecules. Such is the case of hyperglycaemia‐evoked insulin surge and the subsequent rise in plasma leptin that acts on the transient receptor potential melastatin 7 channels of CB glomus cells to raise sympathetic activity and blood pressure (Shin et al., 2019, 2021).

In pathological conditions such as diabetes, hypertension and sleep apnoea, it has been proposed that CB overstimulation may lead to hyperglycaemia and over‐sensitivity to low glucose (Abboud & Kumar, 2014; Gao et al., 2014). Taken together, the available evidence suggests that CBs have a physiological role in blood glucose regulation in both animals and humans. Future studies need to determine whether chronic (intermittent) exposure of the CB to high glucose alters its sensitivity and whether this exacerbates the problems of controlling plasma glucose thereby contributing to diabetes.

## 6 CB AND METABOLIC DISORDERS

The CB serves as a sensor to facilitate activation of counter‐regulatory mechanisms in response to fluctuations in blood O_2_, pH, glucose and CO_2_. The contribution of the CB to the pathogenesis of metabolic diseases such as type 2 diabetes, hypertension and chronic heart failure has been of considerable interest in recent years (McBryde et al., 2013; Pijacka et al., 2016; Ribeiro et al., 2013; Shin et al., 2021). It has been proposed that CB dysfunction causes increases in sympathetic tone and catecholamine levels in the blood; this may significantly contribute to the pathogenesis of diabetes and essential hypertension (Nimbkar & Lateef, 2005). Morphological changes to the CB have been observed in both diabetic and hypertensive patients providing a rationale for possible functional changes in their afferent sensitivity (Cramer et al., 2014). The latter study reported that the CB measured 20–25% larger in patients with diabetes or hypertension or congestive heart failure relative to controls. CB enlargement may reflect production of more glomus cells, fibrosis and/or inflammation; all are potential factors contributing to enhanced CB activity (Felix et al., 2012). However, it remains unclear whether CB enlargement and structural changes are a cause or a consequence of metabolic diseases. Patients with obstructive sleep apnoea (OSA), which is associated with CB dysfunction (hyper‐reflexia) (Peng et al., 2003), have an increased incidence of impaired glucose metabolism and are at increased risk of developing type 2 diabetes; conversely, the majority of diabetic patients also have OSA (Tasali et al., 2008). The chronic intermittent hypoxia experienced by OSA patients can sensitize the CB (Iiyori et al., 2007; Polotsky et al., 2003). Could intermittent hyperglycaemia also provide the conditioning stimulus sensitizing the CB to blood glucose? The CB becomes hyperactive in insulin‐resistant, glucose‐intolerant, hypertensive and obese (fed a high fat diet) animals (Ribeiro et al., 2013; Sacramento et al., 2017) and also in pre‐diabetic patients with insulin resistance (McBryde et al., 2013).

## 7 SYMPATHETIC OVERACTIVITY AND THE SNS: A ROLE FOR THE CB

How might CB sensitization mediate insulin resistance and abnormal glucose metabolism? We propose this is mediated via the SNS. A common factor in cardiometabolic diseases (e.g. hypertension, heart failure, insulin resistance, type 2 diabetes) is chronically elevated sympathetic nerve activity (SNA) (Andrade et al., 2015; Carnethon et al., 2003; Fisher & Paton, 2012; McBryde et al., 2013; Pijacka et al., 2016). The CB is a powerful regulator of SNA, exerting both transient reflex and persistent excitatory drives (Cunha‐Guimaraes et al., 2020). Our group previously demonstrated that hypertension is critically dependent on CB input driving the increased sympathetic tone in the spontaneously hypertensive rat (Limberg et al., 2020; McBryde et al., 2013). Additionally, pre‐clinical and human studies revealed that CB resection significantly lowers both sympathetic vasoconstrictor activity to skeletal muscle (MSNA) and blood pressure in conditions of hypertension and SNA in systolic heart failure (Andrade et al., 2015; Narkiewicz et al., 2016). Given this, the CB may control blood glucose (Gao et al., 2014) through sympathetic regulation of insulin (reduced release either directly or indirectly by reducing blood flow within the pancreas) (Almaça et al., 2018; Prates et al., 2018) and via increased release of glucagon (Wehrwein et al., 2010). This is consistent with the finding that surgical ablation of the CBs prevented the progression of insulin resistance and glucose intolerance in rats exposed to high‐fat diet with pre‐established metabolic dysfunction (Sacramento et al., 2017). In addition, sympathetically mediated vasoconstriction reduces post‐prandial increases in skeletal muscle blood flow impairing glucose uptake into muscles so raising plasma glucose levels and stimulating additional insulin production from the pancreas leading to insulin resistance (Narkiewicz et al., 2016). These reports suggest that heightened sympathetic activity triggered by the CB may be a major contributor to the dysregulation of blood glucose (Nimbkar & Lateef, 2005).

The evidence supports that targeting dysfunctional CBs alleviates diabetes (Nimbkar & Lateef, 2005; Ribeiro et al., 2013) and this appears to be due, in most part, to reduced sympathetic activity. Although CB resection has provided proof of concept data, the CB serves many other important physiological functions that are critical for other homeostatic mechanisms that would also be lost by its denervation/resection. For example, in heart failure patients with sleep apnoea, blood oxygen desaturations were more pronounced and longer following bilateral CB removal, which categorically indicates that their resection is not safe (Niewinski et al., 2021). Bilateral surgical ablation of the CB performed in asthmatic patients or during neck tumour surgery causes permanent abolition of the ventilatory response to hypoxia but it does relieve dyspnoea. In addition, this condition causes a decrease in the CO_2_ sensitivity of the respiratory centre and, in some cases, long‐term resting hypoventilation and hypercapnia (Dahan et al., 2007). To advance beyond CB resection/denervation, there is a need to explore novel pharmacological approaches to target the CBs. Our working hypothesis is that there will be novel druggable targets effective for treating both diabetes and hypertension simultaneously in diabetic patients. Indeed, new pharmacological tools need to be developed to inhibit CB activity in diabetes without affecting its role in other homeostatic processes. Thus, we have actively sought to determine novel targets in the CB that may be involved in blood glucose homeostasis beyond insulin receptors.

## 8 FINDING NOVEL TARGETS IN THE CB AS PUTATIVE TREATMENTS FOR DIABETES AND HYPERTENSION

The global mortality rate (1.5 million per year) and economic burden ($1.3 trillion in 2015 to $2.2 trillion by 2030) of metabolic diseases continue to increase (Bommer et al., 2018; World Health Organization, 2022), emphasizing the need for novel and more effective therapeutic approaches. These are likely to have distinct mechanistic actions different from the existing anti‐diabetic treatments. Advances in diabetes medicine, such as anti‐diabetic oral medications, incretin therapies and novel insulin formulations, are all available, but there remains an unmet need as diabetes therapy proves suboptimal. A clinical trial with 97,852 patients with type 2 diabetes from 11 countries reported that despite anti‐diabetic treatment, only 17% of these patients achieved an HbA1c level <53 mmol/mol (7%) (Kong et al., 2020). In addition, many of those on current treatment have uncontrolled blood glucose levels indicating inadequate or suboptimal therapy (Holman, 1998) and diabetic patients are typically resistant to anti‐hypertensive treatment (Sim et al., 2013). Recently, inhibitors of sodium–glucose co‐transporter 2 (SGLT2) (gliflozins) have been used as anti‐diabetic agents to reduce blood glucose by preventing kidneys from reabsorbing glucose back into the blood (Ansary et al., 2019; Cianciolo et al., 2019). Clearly, SGLT2 blockers provide a new clinical approach but they do *not* ‘cure’ diabetes – the chronic benefits have not been established, and logically, it is not recommended for use in diabetes with renal disorders (Cianciolo et al., 2019). Moreover, SGLT2 blockers treat the symptoms and do not address the fundamental cause. To date, current medications for diabetes do not reduce sympathetic activity, which we argue agonizes diabetes (Smith et al., 2004). New types of treatments for diabetes should include a lowering of SNA, and blood pressure in cases where hypertension coexists as has been suggested in obesity‐induced diabetes and hypertension (Shin et al., 2019, 2021). Targeting the CBs would offer this potential and might add significant advantages to diabetic patients for providing long‐term control of blood sugar. But, how might the CB be targeted pharmacologically in diabetes?

## 9 CB: A NODE OF MULTI MODAL RECEPTORS

It is widely accepted that CB chemoreceptors evoke reflex responses affecting haemodynamics, renal, gastrointestinal, endocrine and metabolic function. To orchestrate the appropriate response, we proposed there are distinct CB afferent pathways connected to selected motor outputs driven by specific CB stimulants/receptors (Zera et al., 2019). Thus, the CB has diverse types of receptors located on different subsets of specific glomus cells or glomus cell clusters.

As discussed above, the pathophysiology of the metabolic syndrome relating to an overactivation of the SNS remains incompletely understood (Tentolouris et al., 2006). Emerging evidence suggests that the CB is not only an O_2_, CO_2_ and pH sensor, but rather a multimodal sensor, responsive to glucose, insulin and leptin and containing large numbers of genes expressing G protein‐coupled receptors; whether these permit the distinct reflex signalling pathways we hypothesize remains unclear (Zhou et al., 2016). We propose that the next step is to find novel receptors in the CB that may act as an interface between raised glucose and the SNS. Discovery of such targets may allow disease‐specific pharmacological intervention to improve outcome in metabolic disease (Figure 2) as has recently been proposed for the transient receptor potential melastatin 7 channel (Shin et al., 2021).

## 10 CB SENSITIVITY GOES BEYOND GLUCOSE AND INSULIN

Using transcriptomics, we have discovered a number of receptors within the CB that may well play a (indirect) role in the regulation of blood glucose via the SNS; these include glucagon‐like protein 1 receptor (GLP1R) and the leptin‐mediated melanocortin pathway – the melanocortin 4 receptor (MC4R) (Pauza et al., 2022; Thakkar et al., 2021).

**FIGURE 3:**
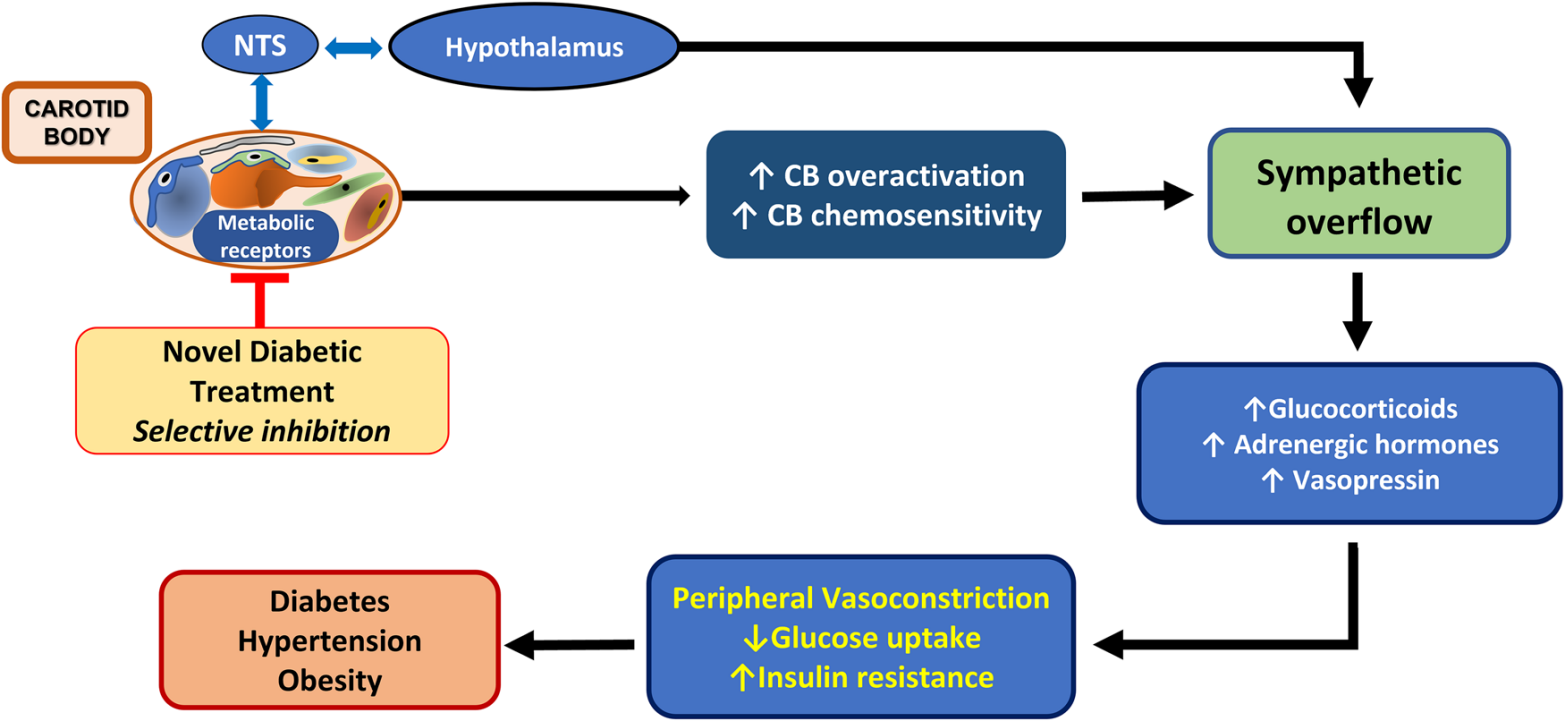
Metabolic syndrome accentuated by the carotid body (CB). Metabolic signals such as insulin, α‐MSH and leptin activate the CB, and its sensory information is relayed to the hypothalamus via the NTS, which triggers elevation in sympathetic activity controlling metabolic homeostasis. Overactivation of CB generates excessive sympathetic activity leading to metabolic imbalance – diabetes and hypertension. Targeting metabolically linked receptors selectively within the CB may be a strategy for the treatment of cardio‐metabolic diseases. NTS, nucleus of the solitary tract.

We propose that both GLP1R and MC4R have a central role in the functional remodelling of the CB in glycaemic control and thus offer potential interventional access for regulating blood glucose (Figure 3). Beyond the CB, these ligands and receptors play a pivotal role in the regulation of energy homeostasis. Glucagon‐like protein 1 (GLP1) is an endogenous peptide that in response to the ingestion of food is secreted into the blood from both intestinal enteroendocrine L‐cells and pro‐glucagon‐expressing neurones in caudal regions of the nucleus of the solitary tract located in the dorsomedial medulla oblongata (Gribble et al., 2003; Jin et al., 1988). Thus, blood‐borne GLP1 could stimulate GLP1Rs located in the CB. Recent studies have established that GLP1R agonists have multiple synergistic effects on glucose‐dependent insulin secretion pathways of the pancreatic β‐cells thereby increasing insulin and decreasing glucagon secretion (Perry & Greig, 2003). Regarding MC4Rs, leptin stimulates POMC neurones in the ARC nucleus of the hypothalamus (Fioramonti et al., 2017). In turn, these neurones release α‐MSH, which stimulates downstream receptors located on PVN neurones that decrease food intake and increase energy expenditure including blood glucose (Barsh et al., 2000). PVN neurones also increase sympathetic activity (Cone, 2005), which may play a role in obesity‐induced hypertension linked to melanocortin pathways. As a POMC‐derived hormone, α‐MSH (an activator of MC4R pathway) released into the bloodstream (Mains & Eipper, 1979; Møller et al., 2016) may also stimulate MC4Rs in the CB, which could engage it in glucose homeostatic mechanisms via autonomic and endocrine pathways.

**FIGURE 4:**
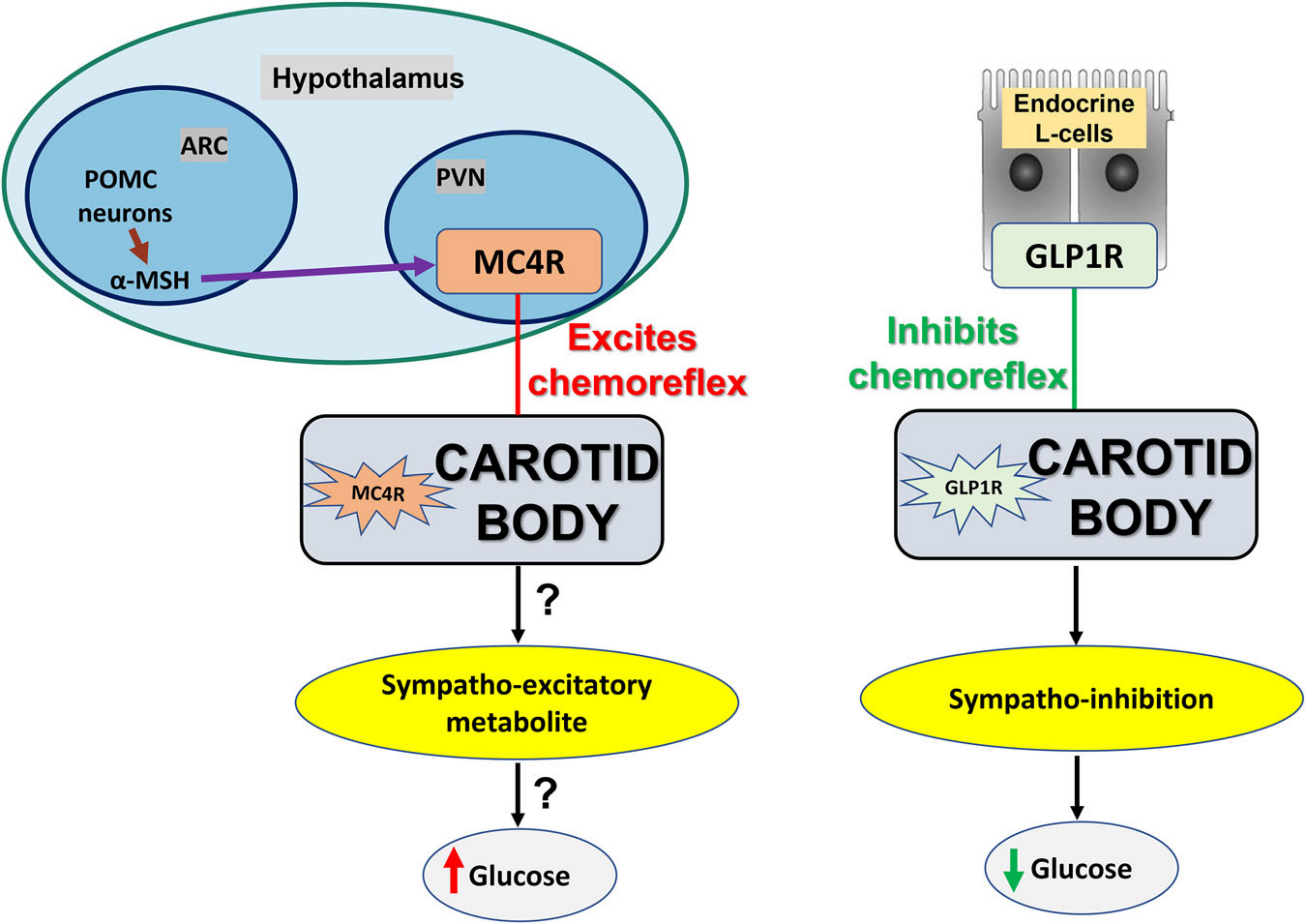
Carotid body is a potential target for controlling cardiometabolic homeostasis via GLP1R and MC4R pathway. Stimulating GLP1R in the CB inhibits the sympathetic output; whether this reduces blood glucose is under investigation by us. In contrast, stimulating the MC4R pathway in the CB may potentiate the sympatho‐excitatory and metabolite pathways to increase blood glucose level. Thus, antagonizing MC4 receptors may suppress blood glucose in conditions of hyperglycaemia. ARC, arcuate nucleus; GLP1R: glucagon‐like peptide type‐1 receptor; MC4R, melanocortin type‐4 receptor; POMC, proopiomelanocortin; PVN, periventricular nucleus.

We propose the testable hypothesis that GLP1Rs in the CB decrease SNA, and MC4Rs in the CB increase SNA, and that these changes in SNA contribute to glucose homeostasis. However, whether these receptors are expressed differentially in the CB in metabolic disease is unknown but an interesting concept due to their close association with glucose regulation. Our recent study using the working‐heart–brainstem preparation revealed that focally administering GLP1R agonist into the CB via close arterial injection inhibited both basal SNA and CB reflex‐evoked SNA (Pauza et al., 2022). Importantly, we show that in response to hyperglycaemia, the chemoreflex hyper‐reflexia and raised basal sympathetic activity can be normalized by GLP1R stimulation within the CB. We also demonstrated in vivo that injecting a GLP1R agonist systemically reduced blood pressure and inhibited the chemoreflex‐medicated increase in blood pressure. This suggests that sympathetic activation post‐prandially may be modulated by GLP1 acting on the CB (Pauza et al., 2022). This ground‐breaking work connects the common pathways between the gastrointestinal tract and the CB and provides a common link between blood pressure and blood sugar control (Figure 4). However, downregulation of GLP1R and upregulation of MC4R in the CB in animals with metabolic dysfunction can be the key factor to the physiological relevance of SNA regulation which is currently undergoing investigation.

## 11 CONCLUSIONS AND FUTURE PERSPECTIVES

Glucose sensing by the brain and pancreas is of critical importance for maintaining metabolic homeostasis. However, the numerous signalling pathways recruited to regulate glucose homeostasis in euglycaemia continue to expand with addition of leptin, ghrelin, α‐MSH and GLP1 to insulin, glucagon and adrenaline. The CB may also contribute to glucose homeostasis as well as hyperglycaemia in diabetes. We believe that metabolically linked pathways within the CB drive peripheral chemosensitivity for glucose homeostasis in health and metabolic disease. As CB overactivation is closely linked to sympathetic hyperactivity, which is known to exist in diabetes and hypertension, manipulating the CB to control sympathetic activity may provide a novel therapeutic strategy to control both high blood glucose and high blood pressure simultaneously. Further investigation of pharmacological interventions in the CB via agonizing and/or silencing distinct metabolic related receptors such as those for GLP1 and MC4 is now warranted. We conclude that the CB is a peripheral convergent node that integrates information on metabolic and cardiovascular status, representing a nodal target for therapeutic intervention in chronic complex diseases. The recent discovery of GLP1Rs and MC4Rs in the CB opens opportunities to better understand the role of this multi‐modal sensor in control of blood sugar in health and disease.

## AUTHOR CONTRIBUTIONS

Pratik Thakkar drafted, revised the review article and prepared the figures; Julian F. R. Paton provided constructive feedback, some new ideas and edited the text, and approved the final version of the manuscript; Audrys G. Pauza and David Murphy provided feedback on the review. All authors have read and approved the final version of this manuscript and agree to be accountable for all aspects of the work in ensuring that questions related to the accuracy or integrity of any part of the work are appropriately investigated and resolved. All persons designated as authors qualify for authorship, and all those who qualify for authorship are listed.

## CONFLICT OF INTEREST

None declared.
